# Biological Relativity Requires Circular Causality but Not Symmetry of Causation: So, Where, What and When Are the Boundaries?

**DOI:** 10.3389/fphys.2019.00827

**Published:** 2019-07-18

**Authors:** Raymond Noble, Kazuyo Tasaki, Penelope J. Noble, Denis Noble

**Affiliations:** ^1^Institute for Women’s Health, University College London, London, United Kingdom; ^2^Department of Physiology, Anatomy and Genetics, University of Oxford, Oxford, United Kingdom

**Keywords:** biological relativity, downward causation, circular causality, entangled causation, boundaries in physiology

## Abstract

Since the Principle of Biological Relativity was formulated and developed there have been many implementations in a wide range of biological fields. The purpose of this article is to assess the status of the applications of the principle and to clarify some misunderstandings. The principle requires circular causality between levels of organization. But the forms of causality are also necessarily different. They contribute in asymmetric ways. Upward causation can be represented by the differential or similar equations describing the mechanics of lower level processes. Downward causation is then best represented as determining initial and boundary conditions. The questions tackled in this article are: (1) where and when do these boundaries exist? and (2) how do they convey the influences between levels? We show that not all boundary conditions arise from higher-level organization. It is important to distinguish those that do from those that don’t. Both forms play functional roles in organisms, particularly in their responses to novel challenges. The forms of causation also change according to the levels concerned. These principles are illustrated with specific examples.

## Introduction

The principle of Biological Relativity is that, *a priori*, i.e., before performing the relevant experiments, there is no privileged level of causality (Noble, 2012). In multi-scale networks of interactions, as found everywhere in organisms, any parts of a network at any level might affect every other part.

The principle is based on mathematical approaches to understanding biological processes. While the differential (or equivalent) equations represent the dynamics of the components of the system, the initial and boundary conditions represent the historical and contextual (environmental) factors without which no specific solutions to the equations would be possible.

The principle has found many applications in physiology and in other fields of biology. This is not surprising since the mathematical point being made is a necessary one, regardless of whether the components are molecular (genes, proteins, and metabolites), networks (at all levels), cells, tissues, organs, or any other kind of component. Moreover, in practice the principle has been applied many times in physiology even before it was formulated as a mathematical principle. All forms of feedback between levels in biological systems inherently assume the principle. It can therefore be seen as formalizing an idea that has been inherent in physiology, at least since Claude Bernard in the 19th century (Bernard, 1878, 1984; Noble, 2008, 2013), and Walter Cannon in the 20th century (Cannon, 1932) formulated the ideas of homeostasis. Nevertheless, the principle is not limited to the usual interpretations of homeostasis as linear circularity. The regulatory systems in organisms do much more than act like sophisticated thermostats. There are no fixed set-points. There are sets of set-points each of which can vary as the organism seeks to maintain itself. Buiatti and Longo (2013) express this point by using the word homeorhesis in place of homeostasis:

“Biological objects are, as discussed by Waddington, “homeorhetic,” as opposed to homeo-static, in the sense that, during their cycles, they keep changing. Moreover, their onto-phylogenetic path is largely unpredictable, though preserving, as long as possible, the internal coherence of an organism and its relations to the ecosystem. It is unpredictable because of the random effects at each level and of the bio-resonance effects between different levels.”

As our article will make clear, the various levels communicate both randomness and order between each other. We agree therefore with Rosen in *Life Itself* (Rosen, 1991, 2000), that it is the *organization of the organism itself* that constrains the component parts, not the other way round. That organization forms the basis of active agency in organisms (Noble and Noble, 2017, Noble, 2018). One of the aims of this article is to interpret the principle of biological relativity in a more radical way.

The principle also raises many other questions. The aim of this paper is to formulate those questions and attempt to resolve them. Foremost amongst those are questions concerning what is meant by a boundary.

As physiologists we might think that question has an obvious answer. Cells have membranes, tissues have surfaces, organs have shapes with anatomical boundaries, the organism has its outer structure, skin. But where are such boundaries of the great systems of the body, the immune, nervous, circulatory, digestive, respiratory, reproductive, and hormonal systems? Merely to ask the question shows that the answer is not obvious. Anatomy is not necessarily the best basis for defining a functional boundary. To varying degrees, the boundaries used in models are somewhat arbitrary. And even when we can identify an anatomical boundary it is not necessarily the mathematical computational boundary.

As an example of the kind of problem we will address consider the problem faced in modeling the electrophysiology of the heart during the 1980s when processes involving changes in ion concentrations were added to the existing equations for the gating of ionic channels (McAllister et al., 1975). Prior to the DiFrancesco-Noble equations (DiFrancesco and Noble, 1985) this had not been done in any systematic way. Yet it was necessary to incorporate changes in K^+^ concentration in intercellular spaces to understand how these could make a non-specific cation channel conducting both Na^+^ and K^+^ behave like a pure K^+^ channel. The new model was completely successful in achieving this aim. But that was not possible without changing the boundaries of the model. One of us explained this boundary problem in 2012:

“The obvious next step was to develop the McAllister–Noble–Tsien model of 1975 to replace i_K2_ by i_f_. But that was much easier said than done. It took a full 5 years of development. This was because it was not just a matter of replacing one ionic channel mechanism by another. It also involved modeling global ion concentration changes for the first time in an electrophysiological model of the heart, including the intracellular calcium signaling. Dario and I did that because it was necessary to explore fully what we had discovered. We did not know then that we would be creating the seminal model from which virtually all subsequent cardiac cell models would be developed. There are now over a hundred such models for various parts of the heart and many different species^1^.”

Extending biological models is often like tumbling a row of dominoes. Once one has fallen, many others do too. The reason is that all models are necessarily partial representations of reality. The influence of the parts that are not modeled must either be assumed to be negligible or to be represented, invisibly as it were, in the assumed boundary conditions and other fixed parameters of the model. Once one of those boundaries is removed, by extending out to a different boundary, other boundaries become deformed too. In this case, modeling external potassium changes required modeling of the influence of those changes not only on the ion channels already in the model, but also on exchange mechanisms, like Na-K-ATPase (sodium pump) and the Na-Ca exchanger. That, in turn, required the model to extend to modeling internal sodium concentration changes, which in turn required modeling of intracellular calcium changes, which then required modeling of the sarcoplasmic reticulum uptake and release mechanisms. For a year or two it was hard to know where to stop and where to stake out the new boundaries” (Noble et al., 2012) (Page 58).

Even more difficult is the fact that physiological boundaries can be dynamic. When and why they occur are also important questions since it is at boundaries that many of the vital functional processes occur. Recall that the nervous system develops from the embryonic “boundary,” the ectoderm, and in single cell organisms the surface membrane can be regarded as its nervous system. Organisms are open systems, so their boundaries are necessarily where much of the action occurs.

## Definitions

### Biological Relativity

Biological relativity is the principle that there is, *a priori*, no privileged level of causation. The necessary mathematical basis of the principle was first proposed in 2012 (Noble, 2012) when it was categorized as a “theory.” It is better viewed as a principle since it expresses the conceptual point that there is no empirical justification for privileging any particular level.

### Upward Causation

Upward causation is the set of processes by which the lower elements in a system interact and produce changes at higher levels. In differential equation models these processes are described by the dynamics represented by the differential equations themselves.

### Downward Causation

Downward causation is the set of constraints imposed by the higher levels on the dynamics at lower levels through determining many of the initial and boundary conditions. El-Hani and Queiroz (2005) use the term, “Downward Determination”, but they agree that what is involved is something that “can be understood in terms of constraints that the condition of belonging to a system-token of a given kind imposes on the behavior of the components.” The sense of cause we are using includes that of determination. We agree that there are different kinds of causation (Noble, 2016) (pp 176–181). Mossio et al. (2013) also emphasize the role of higher level constraints when they refer to “emergent causal powers exerted as constraints, and we claim that biological systems crucially differ from other natural systems in that they realize a closure of constraints.”

### Initial Conditions

**FIGURE 1:**
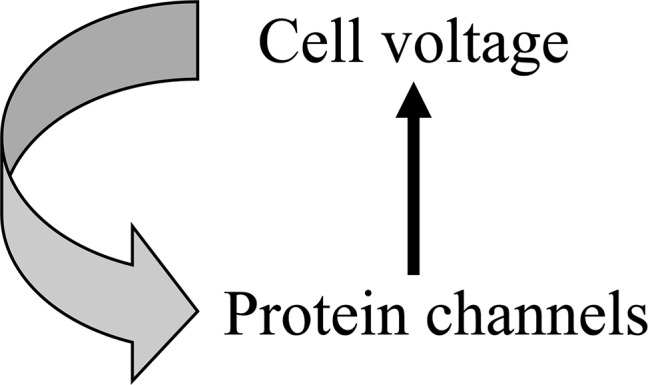
An example of circular causality in physiology. The Hodgkin cycle represents the fact that global cell properties, such as electric potential, control molecular level properties, such as ion channel proteins, which in turn determine changes in cell properties.

Initial conditions are the initial values of each dynamic element at lower levels. They are determined by the history of development of the system, including stochastic variation as well as previous states of the system. The upward and downward forms of causation interact (Figure 1).

### Boundary Conditions

Boundary conditions are the conditions attributable to interaction with the environment. In partial differential equation models these conditions are represented by the state of the spatial boundary of the system. In ordinary differential equation simplifications in which spatial changes are assumed to be instantaneous these conditions are represented by the constant coefficients at any moment in time.

### Structure

Structure is also a condition that could be regarded as initial or boundary according to the modeling chosen.

### Conditioned Causation

Conditioned causation is a state of a system where it would be misleading to attribute causation to any particular element.

## Main Sections

### How Do Upward and Downward Forms of Causation Differ?

The existence of both upward and downward forms of causation is often represented as circular causality. While obviously correct in the sense that both forms exist and, in many ways, must influence each other, such diagrams hide the fact that there is an important difference. The upward and downward forms are necessarily different, just as the initial and boundary conditions of differential equation models are clearly not the differential equations themselves.

It is also important to distinguish conceptual questions about how we see things from what nature does. Nature is a continuum on which we impose somewhat arbitrary boundaries which are dependent on the models we use to understand nature. This point should be borne in mind throughout this article.

#### Upward Causation

Lower levels influence higher levels through the dynamic changes represented by the differential equations. These will result in global changes, for example in concentrations of ions, metabolites, proteins in cells, tissues and organs and these may in turn trigger further changes at any or all of the higher levels.

As an example, consider the processes involved in calcium movements in the various kinds of muscle in an athlete during vigorous exercise. Too much intracellular free calcium may cause maintained serious problems in the athlete’s heart, skeletal muscles or smooth muscles. Any of these, such as a sudden heart attack, may cause severe pain, in turn leading the athlete to collapse. Then the influences become wider and wider as the team coach and physiotherapist enter the scene, which further leads to social interactions. This is an example of unintended effects at a lower level triggering many other events at higher and higher levels.

#### Downward Causation

Now let’s consider how the athlete became an athlete in the first place. He spent hours a day training. This was his decision. It wasn’t a decision of the calcium ions in his muscles, nor of the gene sequences in his DNA. Molecules and ions are not causes in that sense (Noble, 2016). It was a high-level choice that he made (Noble and Noble, 2018) and it resulted in many changes in his musculoskeletal, respiratory and cardiovascular systems, all becoming more powerful. Many of these changes came about through exercise influencing gene expression of the proteins in muscles, the lungs and the cardiovascular system. This in turn changes the innumerable boundary and initial conditions under which all the muscles in the athlete’s body behave. The changes influence how much muscular, breathing and cardiovascular capacity the athlete has. Although the differential equations for each of his muscle fibers will still be much the same, the changed initial and boundary conditions now ensure that the athlete can do the same or even more vigorous exercise without experiencing disabling fatigue and cramp. This is an undeniable physical effect at the molecular level arising from the athlete’s choice of lifestyle. It doesn’t alter the laws of molecular behavior. It alters the solution to the equations for those laws.

#### Identical Twin Athletes

**FIGURE 2:**
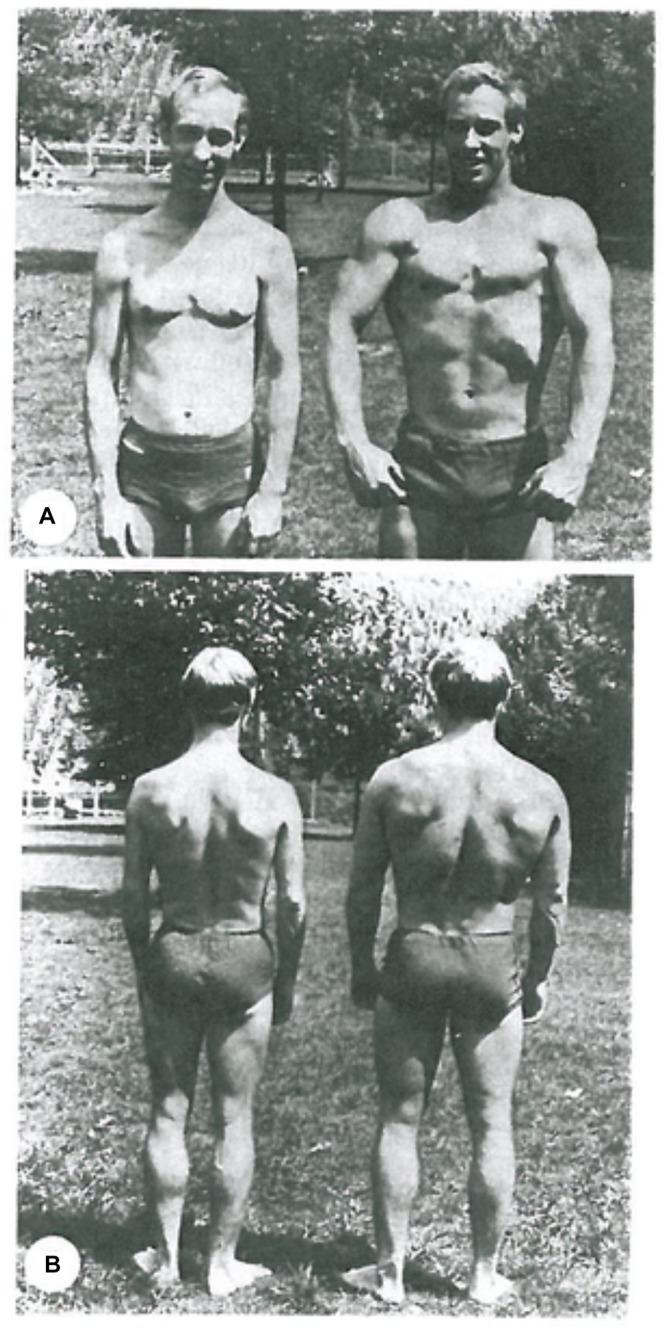
Identical twins. **(A)** Long-distance endurance runner. **(B)** Weightlifter. Notice the highly developed calf muscles in the runner and the contrast with the highly developed arm and chest muscles of the weightlifter. Reproduced with permission from the publisher of Keul et al. (1981).

At this point a rigorous genetic reductionist (Comfort, 2018; Plomin, 2018) might want to argue that no downward causation was involved. The athlete was simply born with the right genes to develop as an athlete. While that must be true – someone suffering from a genetic disease like muscular dystrophy, for example, could not do what the athlete does – it is far from being the complete story. Studies of identical twins who chose very different kinds of sports and exercise training show that very clearly. Figure 2 is taken from such a study (Keul et al., 1981). The runner and the weightlifter showed completely different effects on their body physique. Bathgate et al. (2018) have recently published a more extensive study of many differences in muscle and cardiovascular health and performance in monozygotic twins. They conclude that “the cardiovascular and skeletal muscle systems exhibit greater plasticity than previously thought.” Furthermore they have identified precisely which RNA levels of control are changed by the lifestyle choices.

#### Genome-Wide Association Studies

Genome sequence studies have failed to find just a single or a very few genes that are strongly correlated with athletic performance. A literature search on publications in the period 1997–2014 showed at least 120 genes show correlations with athletic performance, many of the correlations being very small (Ahmetov and Fedotovskaya, 2015). That number of correlated genes is likely to grow as even more extensive GWAS results appear. So much so that some GWAS scientists have come to the conclusion that virtually the whole genome may be correlated with most phenotypes, the so-called omnigenic hypothesis (Boyle et al., 2017). A study of 1520 endurance athletes and 2760 controls “did not identify a panel of genomic variants common to these elite endurance athlete groups” (Rankinen et al., 2016), and see their earlier studies (Rankinen et al., 2000, 2005). One recent study comparing the impact of genes and environment concluded “that the traditional argument of nature versus nurture is no longer relevant, as it has been clearly established that both are important factors in the road to becoming an elite athlete.” (Yan et al., 2016) In a review of elite athletic performance Joyner and Coyle concluded “finding genetic markers that are strongly predictive of either success in endurance athletic performance or somehow preclude it is likely to be a daunting task because of the many cultural and environmental factors that contribute to success in sport, the many physiological factors that interact as determinants of performance, and the heroic nature of the training required” (Joyner and Coyle, 2008).

#### Epigenetic Control

The main reason for the failure to explain athletic performance from genetics alone is that the genome is controlled by the organism and its life-style experiences through extensive epigenetic control.

As an example, athletes have lower heart rates than non-athletes, which was once attributed to greater vagal tone. The changes have now been traced to microRNAs that downregulate expression of the HCN gene, so that the depolarizing current (I_f_) produced in the sinus node cells is reduced by as much as 50% (D’Souza et al., 2017). Moreover, that changes in autonomic tone could not be the explanation was shown as long ago as 1967, but the authors could not at that time identify the mechanism (Sutton et al., 1967). The advent of modern techniques for identifying epigenetic control has transformed this field of study.

The interface between DNA and epigenetic control is therefore another important boundary. It is one of the means by which the organism controls its genome as a “highly sensitive organ of the cell” (McClintock, 1984). This boundary was first identified by Waddington (1957), who was the originator of the term epigenetics. Since then many forms of epigenetic control have been discovered. This control is so effective in transmitting the adaptive properties of the networks that most gene knock-outs have very little effect. The exceptions are, of course, the rare genetic diseases, such as cystic fibrosis, where the networks do not have sufficient plasticity to cope with a knock-out. But, in general, plasticity is common. In yeast, for example, 80% of gene knock-outs are silent in the sense that they produce no phenotypic effect when the yeast is well-nourished (Hillenmeyer et al., 2008). That result has been broadly confirmed by Galardini et al. (2018) who have shown the extent to which the effect of a gene deletion depends on the genetic background. They conclude that “interpretation of the impact of genetic variants on the phenotypes of individuals would likely need detailed gene-phenotype information in more genetic backgrounds than that of a model individual.” We would add that the phenotype background must also be relevant. The boundary between regulatory networks and DNA is necessarily a two-way boundary. The regulatory networks can filter genetic changes, acting as what we have characterized as a “cloud” at the boundary (Noble and Noble, 2017; Galardini et al., 2018).

The downward forms of causation represented by the choices made by the individual organism and the influences of its environment must therefore be widespread and necessary.

### Open Systems and Their Boundaries

One reason why boundaries are important is that all organisms are open systems. The interaction with the environment is an essential process of being alive. It is across the boundary between the organism and its environment that all the exchanges of energy and matter occur. The same principle applies within the organism. There are boundaries between cell components, between cells, tissues, organs, …all the way up. Downward causation can be seen to be traversing a cascade of boundaries. Each level of organization provides the boundary and initial conditions for solutions to the dynamic equations for the level below.

#### Are All Forms of Downward Causation Functional?

So far, we have established why downward causation is effective and that its necessary effectiveness is mathematically demonstrable. Now let’s look at those initial and boundary conditions more carefully. When we inspect the most complete of the mathematical models of skeletal, cardiac and smooth muscles we can identify more than 100 constants in the equations (DiFrancesco and Noble, 1985; Yang et al., 2003; Shorten et al., 2007). Each of those, alone or in combination, reflects an initial or boundary condition. So, there are at least that many parameters that might be sensitive to causative action from higher levels. These parameters are determined by the state of the boundaries between higher and lower levels. In reality there will be many more. The model is just a partial abstraction of reality.

Could all parameter changes in the initial and boundary conditions be attributable to downward causation? There are several reasons why that cannot be true.

##### The lowest boundary: molecular stochasticity

As Robert Brown showed in 1827, fine particles suspended in water show stochastic movement which was eventually shown by Einstein to be produced by random bombardment by individual water molecules. The molecules in cells are an aqueous suspension and must also be subject to Brownian motion. Water, and all molecules, will also be subject to quantum mechanical randomness. On some interpretations of quantum mechanics, all objects are subject to such randomness (Becker, 2018), although it becomes negligible at a large enough scale.

This is a boundary *within* the system. In a sense it is a boundary between levels or scales. Later in this article we will discuss how organisms use this and other boundaries between levels. But here it is sufficient to note that the boundary is fuzzy. There is no precise cut-off scale at which molecular stochasticity, whether quantal or not, becomes negligible. This is a major issue in the interpretation of quantum mechanics (Becker, 2018), but it need not detain us here. We note that it is a good example of a boundary that cannot be given a precise anatomical location. In a sense the boundary is everywhere. It is a boundary between levels of organization.

##### Functional and non-functional initial and boundary conditions

Influences on a system from its environment and higher scales can be of at least two kinds. Some will be contingent and even apparently random. These will provide opportunities for novelty in the organism’s behavior, in much the same way as we have described in related articles (Noble and Noble, 2017, 2018). Stochasticity can be used by organisms to generate novelty. That can happen whatever the origin of the stochasticity, whether molecular within the organism or environmental without the organism.

But what is usually meant by downward causation are influences that arise from the regulatory *organization* at higher levels. Organization is what defines a level as distinct from a scale. Cellular organization defines the level of cells, organ organization defines the level of organs, and so on through the levels.

What do we mean here by organization? What precisely is homeostasis? Yet again, the common diagrams of upward and downward causation can be misleading. Regulatory processes in the body are rarely simple feedback loops maintaining a specific parameter, like blood pressure or temperature, constant. Nor is the circularity a simple feedback loop that can be described as a linear sequence of causation: A leads to B which leads to C and so on. This way of thinking leads to the need to specify the *direction* of the causation, in turn leading to the idea of emergence, usually interpreted to mean that the higher-level organization emerges *from* the lower level activity. But how can that be? At the lower level we can’t even *see* the organization. Low-levels do not possess such organization. The constraints of higher-level organization will be represented by a seemingly disorganized set of initial and boundary conditions. We don’t for example “see” the organization of bird haemoglobins as they vary according to different altitudes by sequencing their genomes. At that level, the different species have used different molecular level solutions to evolve haemoglobins for high and low altitudes. At the functional level, the haemoglobins can be characterized as functional for the altitude at which they live so that all high-altitude birds show higher affinity for oxygen even though the DNA sequences are different (Natarajan et al., 2016). Only at the higher level of organization is the function of the genome changes evident.

We have elaborated on this problem in a previous article (Noble and Noble, 2017). From the molecular level of DNA, RNA, proteins, metabolites, ions etc., we will not be able to see the organization. As we noted earlier, it was not the athlete’s calcium ions that caused his decision to be an athlete.

##### Emergence – a-mergence?

For these reasons, we have argued elsewhere for replacing the term e-mergence (suggesting privileging upward causation) with the neutral term a-mergence (Noble and Noble, 2019). In terms of causation, this requires replacing the linear sequence A causes B which causes C etc., with the existence of the state X, the occurrence of which means that A, B, and C etc., will also occur. This is the characteristic of high-level attractors. Once they occur, they take over the organization of the system. This fact becomes hidden when we insist on a linear causation viewpoint. Yet it is implicit when we solve model differential equations numerically since all factors are taken into account at each integration step. In a cell model we don’t, for example, first calculate the influence of all the global cell parameters (such as potentials and concentrations) and then calculate the influence of the microscopic elements (such as transporter and enzyme states) separately.

This issue of simultaneity of action is fundamental. Another way of expressing it is to ask whether circular causality can be said to have a direction. Diagrams often strongly imply that they do, by giving the impression that, if one could be a nano-level observer, one would see one stream of causation running upward and another flowing downward. That picture is far from the reality. This is where the mathematical interpretation of circular causality is so useful in providing a totally different picture of the situation, since the integration procedures must proceed simultaneously (Noble, 2012). A nano-level observer would surely see something more like a cloud of happenings, which would not be resolvable into separate streams of happenings^2^.

In this respect, the Biological Relativity interpretation of multi-level causality resembles wave theories of quantum mechanics. Electrons circling a nucleus, for example, are referred to as a cloud because the wave interpretation does not, and cannot, identify where any particular electron may be. The cloud exists as a quantum mechanical state that is precisely and quantitatively described by quantum mechanical wave equations. What matters is the existence of that state, not where any particular electron may be.

Similarly, it is the *state* of a multi-level biological system that matters, not just its breakdown into any particular separate sequences of causation. In any case everything else depends on the existence of the combined state of the system, which is unresolvable into two streams of causation. Not only would there not be two separate streams of causation, what is happening would not be evident in a single slice in time. The attractor or any other organizational property would only be apparent in a phase space representation within which the organizational pattern can be appreciated in an extended time period.

Purely reductionist thinking tends to avoid such language, which is usually criticized as being somehow fuzzy. But it is no more so than quantum mechanics. The analogy is quite close, since the breakdown of an attractor state can be viewed in much the same way as the collapse of a QM wave function. The same criterion for success is also applicable: is the resulting theory empirically predictive? Multi-scale physiological modeling is increasingly successful by this criterion. Vecchi et al. (2018) have introduced the term Entangled Causation to represent their conclusion that “there is no biological rationale for assuming that every switch point should be regulated by a single causal factor and that development generally involves interactive causation in the form of multiple simultaneously contributing difference-making causes to the regulation of the threshold mechanism at every switch point.” The resemblance of their conclusions to ours is clear.

Representing organisms as high-level attractors and similarly organized states therefore corresponds much better to what we know experimentally. Most changes at the level of DNA are buffered by the high-level attractors. As Baverstock and Rönkko have shown, the phenotype can best be “represented by high dimensional attractors, evolutionarily conditioned for stability and robustness” (Annila and Baverstock, 2014; Baverstock and Rönkkö, 2014).

##### Further Physiological examples

We have already used a specific example, that of muscular exercise, to illustrate some of the main points of this article. We will now give further physiological examples. These will illustrate the variety of the forms of boundaries in physiology. It will be through understanding this variety that we will be able to summarize some general principles in See Sections “Delayed differential equations” and “Boundaries between levels: how do they differ?”

###### Anatomical and functional boundaries in the heart

The heart as an organ has many anatomical boundaries within it since the cells from the sinus node, the atrium, the AV node, the ventricular conducting system, and the ventricle all have different electrophysiological properties, which reflect different protein expression patterns. These in turn are susceptible to different dynamic states within the regulatory networks. The anatomical boundaries between these parts of the heart will therefore experience different magnitudes and direction of ion current flow between them.

These differences also occur within each area. Ventricular cells, for example, differ between epicardial cells and endocardial cells and between the base and apex of the ventricle. These differences are very important in the interpretation of the electrocardiogram. Cells within the sinus node also differ in a graded way. Cells from the periphery have a higher natural frequency than cells near the center.

These differences led to a surprising result when multicellular models of the sinus node became possible, as a result of the increase in computer power offered by the first parallel computers in the 1990s. Using a 64,000 parallel array with each computer processor representing a single cell model, it was found that the origin of the heartbeat, defined as the first cells to depolarize, occurred at the periphery of the model node, creating a wave that spreads inward toward the center (Winslow et al., 1993). This is surprising since in a real heart the beat originates near the center and spreads outward toward the periphery.

The solution to this puzzle was given by the experimental work of Boyett et al. (2003). When the sinus node is carefully separated from the atrium by surgical dissection, the node does indeed behave like the computer model. The sinus-node/atrium boundary is therefore functionally important in creating the conditions in which the beat begins toward the center of the SA node. The high negative resting potentials of the atrial cells together with their high membrane conductance due to high expression of inwardly rectifying potassium channels create the functionality of the complete structure.

Furthermore, the shape of the boundary involved here is not a simple circle or ellipse. The regions of atrial and sinus cells interdigitate in a pattern that enables the weak sinus cells to succeed in depolarizing the stronger atrial cells by almost entirely surrounding cells at the tips of the interdigitations. The impedance-matching process at this boundary is critical in enabling the SA node signal to succeed in spreading through every part and so exciting the whole heart in a functionally important sequence. This functionality is clearly constrained by the high-level geometric structures (Boyett et al., 2003).

###### Intercellular potassium waves generate oscillatory growth patterns in bacterial films and in vertebrate circulations

Not all bacteria are free swimming single cell organisms. Many form multicellular colonies in the form of films, strings and various matted structures. In their patterns of growth these colonies can behave as intercommunicating networks resembling those of multicellular organisms. Thus, a bacterial film may not grow at a constant speed. It may instead display oscillations in growth rate. These oscillations have been shown to be produced by communications between the cells involving intercellular potassium waves. In effect the cells at the center of the colony are informing those at the periphery when to divide since the release of potassium ions is linked to metabolic activity which in turn enables division to occur (Prindle et al., 2015). Prindle et al. (2015) conclude: “The ensuing “bucket brigade” of potassium release allows cells to rapidly communicate their metabolic state, taking advantage of a link between membrane potential and metabolic activity. This form of electrical communication can thus enhance the previously described long-range metabolic co-dependence in biofilms” (Liu et al., 2015).

###### Intercellular communication is widespread even in nominally single cell organisms

Potassium wave communication occurs in many organisms, particularly in the circulation in vertebrates, where it is responsible for functionally important phenomena like retrograde vasodilation (Longden et al., 2017). The evolutionary origin of such communication between cells and tissues is clearly very ancient.

Such boundaries can be maladaptive. In the brain, the phenomenon known as spreading depression is due to the generation of a wave of potassium efflux arising principally from glial cells that leads to the depolarization of neurons, resulting in their refractoriness to the nerve impulse with consequent loss of neural activity.

In such forms of communication, the boundaries are fuzzy and distributed. What is a component from some levels may be a boundary at others. Functional boundaries can come and go according to the state of the whole system. Boundaries are themselves therefore interactive. Thus, in the life history of Amoeba *Dictostylium* (?), intercellular boundaries exist at some phases of the cycle and not at others since the organism can function either as an integrated well-ordered colony or as single cells or spores.

###### Cancer formation and suppression

The standard theory of cancer formation is the somatic mutation theory according to which the accumulation of mutations cause some cells to proliferate abnormally to develop the cancerous tissue. A competing theory is the tissue organization field theory which attributes the cause of cancerous development to properties at a tissue rather than cell or genetic level (Soto and Sonnenschein, 2011). This theory locates the main action at the boundaries between individual cells and the state of the surrounding tissue. A key prediction of this explanation of cancer is that cancers may be “normalized” by changing the boundary, i.e., by transplanting the cancerous or precancerous tissue into normal tissue. This has been shown to happen (Mintz and Ilmensee, 1975; McCullough et al., 1997; Maffini et al., 2005; Kasemeier-Kulesa et al., 2008).

###### Sponges

All multicellular organisms and colonies of unicellular organisms face the problem of the open boundary requiring exchange with the environment. If the cells are packed too close together some will not be able to exchange nutrients and waste rapidly enough. In See Section (“Intercellular Potassium Waves Generate Oscillatory Growth Patterns in Bacterial Films and in Vertebrate Circulations”) above we saw that bacterial colonies solve this problem by signaling when parts of the colony experience metabolic stress. Sponges solve this problem in a different way: the organism is structured using collagen forming open networks of spaces through which freshwater or seawater can flow. Water is wafted through the channels by flagella on the lining of cells, so enabling all cells to exchange freely with the environment. This movement of fluid is the sponge’s equivalent of a circulation. There is experimental evidence that this slow-moving aqueous boundary enabled the earliest animal sponges to survive in very low oxygen levels and therefore to evolve before the general oxygenation of the environment around 580 million years ago (Mills et al., 2014).

##### Delayed differential equations

Equations of this form are sometimes used to represent situations in which there is a significant delay in the action of a part or level of the system on its components (Bocharov and Rihan, 2000). These are important because they also show that chaotic behavior can arise from deterministic equations (Ikeda and Matsumoto, 1987). This form of mathematical representation may seem to contradict our earlier claim of simultaneity of upward and downward causation. That this is not so can be understood by noting that such equations represent an *ordinary* differential equation simplification of any real system, where a full representation would require *partial* differential equations in which the delay would be modeled as a diffusion process in space. This more complete representation would then satisfy the simultaneity condition, with the delay being properly computed in time at each point in space. At each point in space there would be no delay.

##### Boundaries between levels: how do they differ?

**FIGURE 3:**
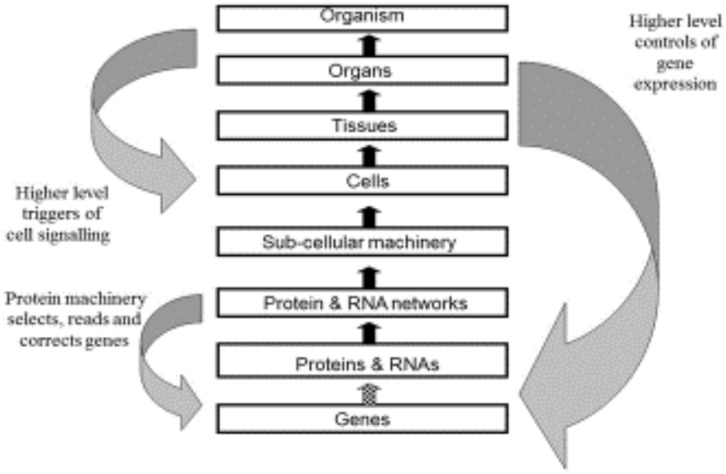
Original multi-level causation diagram illustrating some of the forms of downward causation. Redrawn from Noble (2006), Figure 2.

Figure 3 shows the original diagram of multi-level causation (Noble, 2006). The downward arrows were drawn as large and as separate arrows to emphasize the importance of downward causation [see also (Tasaki, 2013)]. These are the forms of causation that constrain the lower levels and which are necessary for an organism to be alive.

However, there are two aspects of this diagram that could be misleading.

First, both the upward and downward forms of causation differ in their details as we move between the levels. We have discussed examples of these differences in the present paper. An important difference that we will highlight here is the difference between the downward forms of causation onto the genome. The arrow between *Protein and DNA Networks* and *Genes* (the smaller left downward arrow) will consist of molecular details concerning the set of transcription factors, regulatory RNAs and methylation by which molecular events at the network level control gene expression. The higher level causation of the same process (right downward arrow) will include properties at the highest levels of the organism that would enable these controls of the genome to be understood functionally, for example why some cells are constrained to produce the patterns of expression for bone cells while others are constrained to become heart cells, albeit from the same genome. Comparable differences occur between the upward arrows. The arrow from *Genes* to *Proteins and RNAs* consists in the transcription and translation machinery of cells. That between *Cells* and *Tissues* consists in the processes that bind cells together to form tissues. The causation at the different levels depends on all the other forms of causation between lower and higher levels. There is a form of nesting of causation, both upward and downward.

Second, as we have already shown, it would be a mistake to think of the upward and downward causations between any levels as sequential, with one occurring before the other. The lesson we learn from representing these forms of causation in mathematical models is that they are necessarily simultaneous.

**FIGURE 4:**
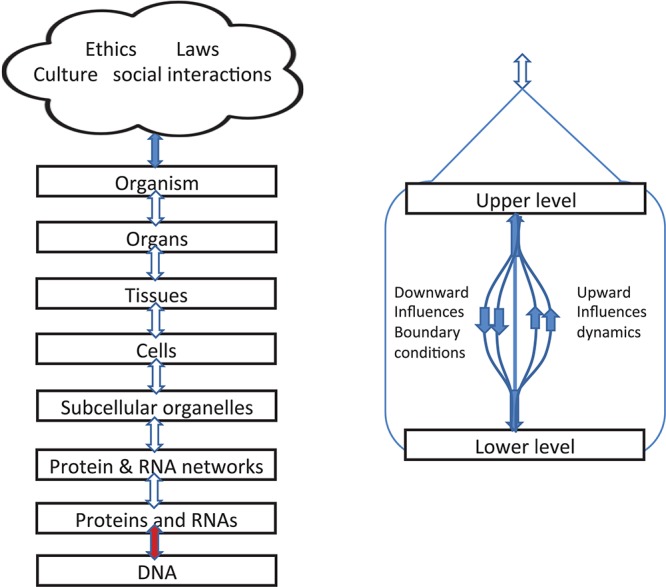
Left: Representation of levels of interaction emphasizing that upward and downward causation operate simultaneously and are shown as double arrows. Right: diagram showing that, within each bidirectional causal arrow, there are different forms of causation, up and down.

Figure 4 gives a different representation in which double-headed arrows are used on the left to indicate the simultaneity of action between the different levels. Yet it is still formally correct to say that each of these consists of different kinds of causation. Some will be stochastic, others are ordered constraints. We can therefore imagine these as formally separate lines, as illustrated on the right hand diagram.

The brown colored arrow between DNA and the level of proteins and RNAs is special. The upward influence is a kind of template: genes as DNA sequences act as a template for amino acid sequences in proteins. The downward influences are twofold:

###### Normal

Influence on expression levels of proteins and RNAs with no change in DNA sequence.

###### Special

Creation of new DNA by, e.g., the immune system, and other forms of targeted mutations and natural genetic engineering.

##### Boundaries beyond the organism

Figure 4 also illustrates the fact that, since organisms are open systems, there are necessarily levels above that of the whole organism, extending into the various forms of social interactions and, in the case of humans, the constraints of laws and ethics. Here we simply note that they also introduce different forms of causation, including constraints on behavior exerted by reasons and habits. The blue arrow at the top therefore represents the very different forms of causation that depend on reasons and contextual logic The relations and distinctions between reasons and causes are deep philosophical issues which we do not deal with here. This is part of the reason why we have represented the social and cultural factors involved all together as a single cloud. The diagram does not imply fuzziness or “ghostliness” in the actions on organisms. On the contrary, there is nothing ghostly about the fact that choice of lifestyle affected the muscles of the identical twins in Figure 2 so differently, nor in the fact that Bathgate et al. (2018) have now identified the specific RNA changes involved at the molecular level.

This is a suitable point to comment on Craver and Bechtel (2007) case against the use of “causation” in top-down influences. Their case is that “the notion of top-down causation is incoherent or that it involves spooky forces exerted by wholes upon their components.” We see nothing incoherent in the expression of top-down influences in terms of boundary and initial conditions. Open systems necessarily have boundaries. The forms of causation across those boundaries differ in the two directions, as we have shown and acknowledged throughout this article, but they are nonetheless real. Both forms are mathematically rigorous. As differential equation models show, they are both also necessary. An important clue to the substantial difference between our viewpoints is their statement that “both phrases describe *mechanistically mediated effects*” (their emphasis). We agree that setting boundary conditions is not “mechanistic” in the same sense as the dynamic role of upward causation represented in the differential terms in model equations. Moreover, processes that harness stochasticity are not well represented by the term “mechanistic.” It is precisely their non-mechanistic nature that is important.

We are not the first to draw attention to the fact that the causal effects of organization at higher levels are exerted through the boundary conditions at lower levels. The physical chemist Michael Polanyi made exactly this point as long ago as 1968 (Polanyi, 1968):

“Therefore, if the structure of living things is a set of boundary conditions, this structure is extraneous to the laws of physics and chemistry which the organism is harnessing. Thus the morphology of living things transcends the laws of physics and chemistry.”

Polanyi’s article is remarkably close to our use of differential equation models to illustrate the different forms of causation in multi-level interactions. The only aspect of his work that has dated is his complete acceptance of Watson and Crick’s Central Dogma. He wrote “the morphogenetic process is explained in principle by the transmission of information stored in DNA.” He did not know that organisms can influence DNA sequences (the downward aspect of the brown arrow in Figure 4) and that much more than DNA is involved in the morphogenetic process.

It is difficult to represent all of these important theoretical distinctions in a single diagram. Figures 1, 3, 4 in our article should therefore each be taken as partial guides to understanding. They each have their limits in representing the conceptual distinctions we are making.

## Discussion

### The Questions in Our Title: What, Where and When Are Boundaries?

#### What?

Our paper shows that there are many kinds of boundaries in and around living organisms. Furthermore they are not usually, or ever, passive. They are an essential ingredient of functionality. The reason is that organisms are open systems, operating far from equilibrium. Boundaries are where many of those non-equilibrium processes take place. We cannot therefore understand the behavior of organisms or their parts from their composition alone, and certainly not from the genome alone. The consequences for physiological research are profound. Isolated components of organisms, whether molecules, cells, tissues or organs, do not necessarily behave in the same was as those components *in situ*. This fact is evident even at the molecular level. Proteins, for example, assume different forms in different environments (Balchin et al., 2016) and so do the processes in which they take part (Garcia-Contreras et al., 2012).

#### Where?

In answering this question we need to remember that it is we who decide what to study in physiological research, whether whole organisms or their components. The way in which we divide nature up determines where the boundaries lie in modeling systems. Where a boundary exists therefore depends on our choice (see the example of the DiFrancesco-Noble equations cited in the Introduction). These choices are not arbitrary, they depend on what has already been discovered. As an example, before the discovery of the variety of epigenetic controls of the genome, the idea of a boundary between the genome and its control by cellular and higher level processes would not have been conceivable. The discovery of these processes and the relevant boundary has far-reaching consequences for physiological research, including interpretations of the Central Dogma of Molecular Biology and of the Weismann Barrier (Noble, 2018).

Choice of boundary also plays a major role in the way in which multi-scale physiology discovers the relative importance of different molecular components. Examples in this article include how the extensions of heart muscle modeling in the 1980s led to the discovery of the quantitative importance of the sodium-calcium exchanger, and how the importance of this exchanger and its regulation has now been discovered using a similar shift from cell to tissue level modeling in skeletal muscle.

#### When?

Organisms develop, so many boundaries do not exist in the same way at the earliest, single cell, stages. Furthermore, they may differ in their ingredients from system to system even though achieving similar objectives. Boundaries between levels can obviously only arise when those levels develop.

#### Clarifications of the Principle of Biological Relativity

Our article clarifies several aspects of the Principle of Biological Relativity.

(1) The forms of causation involved in downward and upward causation are fundamentally different. Downward causation consists in constraints exerted by higher levels on the initial and boundary conditions within which the dynamics of lower level elements operate. By contrast, upward causation is the way in which those dynamics influence higher level states.
(2) These two forms of causation do not form a temporal sequence. They occur simultaneously.
(3) It is the state of organization of a higher level that can constrain lower levels. Causation by a state means that it does not make sense to separate out causation by any one element of the state.
(4) Conditioned causation exists in attractors since any perturbation of the state will be resisted. The strength of an attractor can be measured by the speed with which it re-establishes itself (Kaneko, 1998). The strength of downward causation in organisms is generally high since organisms are very effective at resisting changes in phenotype in response to changes at the molecular level, including changes in DNA sequences. Some authors describe conditioned causation as entangled causation (Vecchi et al., 2018). This is a term borrowed from quantum mechanical theory. The analogy is correct to the extent that the causal states involved should not be separated and the entanglement involved resembles that in quantum mechanical states. But there is also an important difference, which is that entangled states in quantum mechanics are very fragile, collapsing in a fraction of a second, whereas the attractor states in biology are often very robust.

#### Consequences for the Foundations of Physiology

(5) By clarifying the principle of biological relativity, and the nature of the boundaries, multi-level physiology gains rigor. We have not used specific mathematics in this article, nor are many of the points we have discussed primarily mathematical. They are points about the fundamentals of *physiology*. Expressing those fundamentals in terms of arguments drawn from mathematics simply shows that they can, in principle, be as rigorous as any form of science.
(6) What have we not explained? We believe our article opens up many further questions concerning the nature of multi-level physiology. In See Section “Boundaries Beyond the Organism” we have drawn attention to the fact that the causal relations between different levels differ in important ways. One of the most important of these is the increasing role of logic and reasons as we move up to and beyond the level of the whole organism. This is one of the most intractable problems in philosophy and clearly requires more research.

## Author Contributions

All authors listed have made a substantial, direct and intellectual contribution to the work, and approved it for publication.

## Conflict of Interest Statement

The authors declare that the research was conducted in the absence of any commercial or financial relationships that could be construed as a potential conflict of interest.

## Footnotes

1 www.cellml.org

2 In any programming of the integration procedure the precise algorithm used depends on the integration formula used. Usually this consists in successive iterations until a preset level of accuracy is achieved. It would not make sense to divide the integration step up into parts. The step itself is just an approximation to an infinitesimally small step. From the viewpoint of this article everything computed in each step can be regarded as an approximation to true simultaneity.
